# Mapping neurological symptoms and muscle tension representations in impaired gray matter volume of Wilson disease

**DOI:** 10.3389/fneur.2025.1560848

**Published:** 2025-06-05

**Authors:** Yufeng Ding, Kegang Cao, Wenming Yang, Sheng Hu, Jing Zhang, Yulong Yang, Xuran Zhang

**Affiliations:** ^1^Department of Neurology, Dongzhimen Hospital, Beijing University of Chinese Medicine, Beijing, China; ^2^Department of Neurology, The First Affiliated Hospital of Anhui University of Chinese Medicine, Hefei, Anhui, China; ^3^Department of Electronic Engineering and Information Science, Medical Imaging Center, University of Science and Technology of China, Hefei, Anhui, China

**Keywords:** Wilson disease, functional magnetic resonance imaging, dystonia, movement disorder, the Unified Wilson Disease Rating Scale for Neurology

## Abstract

**Objectives:**

Neurodegenerative changes are key manifestations of Wilson disease (WD), causing neurological symptoms including parkinsonism, tremors, and dystonia. However, the neuroimaging correlates of specific neurological manifestations (especially dystonia) in WD remain poorly characterized.

**Methods:**

37 WD patients and 37 healthy controls (HC) were recruited. All subjects underwent structural magnetic resonance scanning, muscle biomechanical measurements, and the Unified Wilson Disease Rating Scale for Neurology (UWDRS-N) assessment. Neurodegenerative changes, identified as gray matter volume (GV) changes, were analyzed via voxel-based morphometry (VBM) in WD compared to HC. Clinical symptoms were linked to GV changes in WD patients’ brains.

**Results:**

Compared with HC, WD patients had GV loss in the bilateral caudate nucleus, putamen, cerebellum (Crus1), left amygdala, right posterior insular lobe, and right parahippocampal gyrus and increased GV in the bilateral anterior insular lobes. In cortical areas, UWDRS-N significantly negatively correlated with GV in the bilateral posterior insula lobes, part of temporal lobe, optic cortex, frontal lobe, and cingulate cortex, while positively correlated with that in bilateral anterior insular lobes and putamen. Moreover, the GV from the left parahippocampal gyrus, bilateral hippocampus, and bilateral caudate nucleus showed a strong positive correlation with the *F* value of the right gastrocnemius medial head.

**Conclusion:**

In WD patients with neurological symptoms, obvious abnormal GV values in the cortico-striatal-thalamo-cortical (CSTC) circuit were noted. These GV changes were linked to UWDRS-N and correlated with muscle tension. The study mapped UWDRS-N and muscle biomechanics in GV-impaired areas, suggesting altered GV (especially in basal ganglia) as a key imaging sign of WD severity. This indicates that the CSTC circuit could act as a biomarker for WD neurological symptoms and affect WD dystonia mechanisms. Additionally, it shows that muscle-related biological parameters can assess WD dystonia severity and neurological damage.

**Clinical trial registration:**

clinicaltrials.gov, identifier NCT05305872.

## 1 Introduction

Wilson disease (WD) is a rare autosomal recessive copper metabolism disorder. It is associated with the *ATP7B* gene mutations. The disease mainly causes toxic copper overload in the liver and central nervous system (1). Neurological symptoms represent one of the most prevalent clinical manifests of WD, comprising tremors, dystonia, and dysarthria (2). Studies show that WD neurological symptoms are predominantly attributable to extensive brain structural damage, particularly within the basal ganglia (3, 4).

The Unified Wilson Disease Rating Scale (UWDRS) is one of the most commonly used scales for the assessment of the clinical symptoms of WD patients (5). Langwińska-Wośko et al. (6) assessed the progression of neurological symptoms in WD patients using the UWDRS. Rędzia-Ogrodnik et al. (7), linked the neurological part of UWDRS (UWDRS-N) scores with magnetic resonance imaging (MRI) manifests, and identified acute and chronic damage in WD. The current study used UWDRS-N to evaluate the severity of neurological symptoms of WD patients. However, the UWDRS-N scale has inherent limitations in quantifying symptom severity. Specifically, its dystonia assessment relies on subjective ordinal ratings (e.g., 0–4 scales) based on clinical observation, which may lack sensitivity to subtle changes and do not incorporate objective biomarkers. This gap underscores the need for complementary quantitative measures to enhance phenotypic characterization in WD (8). Dystonia is a common specific symptom of the nervous system and mainly relies on subjective clinical physical examination, lacking objective quantitative evidence (9). Therefore, this study used the Digital Muscle Function Assessment System, MyotonPRO^®^ (MyotonAS, Tallinn, Estonia) (10) to ascertain muscle tension levels and provide an objective index of the severity of dystonia in WD patients.

Dystonia is a primary component of WD’s neurological manifestations and exerts a substantial impact on WD patients. Clinical studies have revealed that brain structural damage in WD patients is related to their UWDRS-N score (7). Studies have demonstrated neurodegenerative changes in both hepatic and neurologic WD brain, especially extensive brain atrophy in WD with neurological symptoms (11). There is an incomplete understanding of the relationship between damage to different brain regions and the neurological symptoms observed in WD. Our previous study incorporated muscle biomechanical parameters into resting-state functional magnetic resonance imaging (rs-fMRI), suggesting that dystonia in WD may involve neural mechanisms within the lenticular nucleus-cerebellum circuitry (12). However, the mechanism of how dystonia relates to neurodegenerative changes within the brains of individuals with WD needs to be further investigated.

Voxel-based morphometry (VBM) is a commonly used technique in brain morphology studies (13–15). Quantifying local gray matter voxels’ size and signal intensity allows quantitative volumetric analysis of the whole brain for healthy individuals and neurodegenerative patients. The technique is widely used to detect structural changes in brain disease, suggesting its potential role in clinical auxiliary diagnosis and prognostic assessment of brain disorders. In particular, VBM’s ability to pinpoint spatially specific gray matter alterations provides a robust framework for examining WD-related brain damage, which may involve both cortical and subcortical regions and manifest as distinct clinical symptoms.

Here, we hypothesize that neurological symptoms assessed by the UWDRS-N scale are associated with extensive gray matter volume (GV) alterations across cortical and subcortical regions, while elevated muscle tension levels are primarily linked to subcortical structural changes in WD patients. To address this hypothesis, we first employed VBM to compare region-specific GV differences between WD patients and healthy controls (HC). Finally, correlation analyses were conducted to evaluate the relationships between GV changes in identified brain regions and clinical parameters, including UWDRS-N scores and quantitative muscle tension measurements.

## 2 Materials and methods

### 2.1 Participants

From April 2023 to April 2024, 37 patients with WD combined with dystonia and 37 age- and sex-matched healthy controls were recruited from the First Affiliated Hospital of Anhui University of Chinese Medicine (AHUCM) for the study. The inclusion criteria were: Meeting the 2012 European Association for Liver Research diagnostic guidelines for WD (16); being able to perform basic communication and visual, auditory, reading, and writing activities; and being diagnosed with lower limb dystonia according to the relevant dystonia criteria (17). Exclusion criteria included patients with: Pregnancy and lactating; excessive head shaking that interfered with MRI data acquisition; metal implants or a history of cranial surgery; those who had used drugs that could affect dystonia, such as benzhexol or levodopa, within 2 months before the study entry; other conditions that could affect dystonia, such as encephalitis or traumatic brain injury; those with psychiatric disorders or other serious systemic diseases.

The study was conducted in accordance with the ethical standards set forth in the Declaration of Helsinki and was approved by the Human Research Committee of the First Affiliated Hospital of AHUCM (2021AH-60). The results were reported according to the Standard for Reporting Diagnostic Accuracy (STARD) guidelines (18). Prior to enrolment, all subjects were required to sign a written informed consent form.

### 2.2 Clinical and biochemical assessment

The Unified Wilson Disease Rating Scale (UWDRS) comprises three subscales: neurological (27 items), hepatic (9 items), and psychiatric (19 items), totaling 55 items. Each item employs a 5-point scale from 0 to 4 points (0 = asymptomatic; 4 = most severe manifestation), with higher composite scores indicating greater neurological impairment.

Two neurological experts with WD management experience conducted the UWDRS-N assessments. The neurological subscale points was prioritized as it accounts for 49.1% (27/55 items) of the total UWDRS items and demonstrates strong correlation with UWDRS (19). This scale is commonly used to measure the severity of WD (20). We highlight the neurological examination scores (UWDRS-N) to reflect the severity of neurological symptoms. To ensure rating consistency, both evaluators underwent standardized UWDRS training prior to data collection. Discrepancies exceeding 10% in total subscale scores were resolved through consensus discussion.

Dystonia is common in patients with WD (21). It is focal and segmental in the early stages, becoming generalized, usually worsening as the disease progresses, and is often complicated by severe limb spasticity in the later stages (22). The present study focused on lower limb dystonia in light of the aforementioned studies on dystonia and the recent research conducted by our group on upper limb dystonia in WD (23). A digital muscle function assessment system, MyotonPRO^®^, was employed, which is designed to provide a comprehensive evaluation of muscle function (24). The degree of dystonia in the patient’s lower limbs was assessed by measuring muscle biomechanical level parameters. The *F*-value was the main parameter measured, which is the frequency of muscle oscillations and reflects the degree of muscle tension and accurately assess the state of muscle tension (12).

### 2.3 MRI data acquisition

This study used a 3.0 Tesla magnetic resonance scanner (Discovery MR750, GE Healthcare, Milwaukee, WI, United States). During the scan, participants stabilized their heads using sponge immobilisation devices, wore noise-cancelling earplugs to reduce the effect of noise on the subject, closed their eyes, and maintained a supine position without moving during the examination. T1-weighted images were obtained using a T1-3D BRAVO sequence. The principal parameters were as follows: Repetition time (TR) = 8.16 ms; echo time (TE) = 3.18 ms; flip angle (FA) = 12^°^; matrix = 256 × 256; field of view (FOV) = 256 × 256 mm^2^; resolution = 1 × 1 mm^2^; slice thickness = 1 mm; a total of 170 slices were scanned.

### 2.4 Data preprocessing

The structural MRI data were analyzed using the FMRIB Software Library (FSL, Analysis Group, Oxford, United Kingdom) 5.0 version. Subcortical gray matter volume measurements were performed using VBM. The 3D T1-weighted images of all subjects were extracted from their respective structural MRI images and segmented into gray matter, white matter, and cerebrospinal fluid. Gray matter (GM) was highlighted using the FSL Automatic Segmentation Tool (FAST). Linear and non-linear alignment was performed using the alignment toolbox to normalize the segmented GM images to the Montreal Neurological Institute’s 152 (MNI152) standard brain template. The GM images were modulated and smoothed using an isotropic Gaussian kernel with a sigma of 3 mm. All segmentations were checked for visual quality.

### 2.5 Statistical analysis

Statistical analysis was conducted using the SPSS software (version 25.0, IBM Corp., Armonk, NY, United States). The *F* values of muscle biomechanical parameters in the healthy control group and the constant WD group were consistent with normal distribution by the K-S test. A two-sample *t*-test was used to compare the two groups of numerical variables, and *p* < 0.05 was considered statistically significant. A permutation-based non-parametric testing approach with 5,000 random permutations assessed group differences in GV between WD and HC. The significance threshold was established at *p* < 0.001, utilizing the threshold-free cluster enhancement method with family-wise error (FWE) correction to adjust for multiple comparisons. The GVs of WD in cortical regions that exhibit significant GV changes were parcellated into HCP_MMP1.0 atlas (25) and subcortical regions that exhibit significant GV changes were parcellated into Tian’s Subcortical atlas with scale VI (26). The GV in each parcel of the cortex and subcortex was analyzed using Pearson correlation analysis to assess its association with the UWDRS-N score and muscle tension *F* value. The correlation results were corrected for multiple comparisons using the FDR method, with a significance level of *p* < 0.05.

## 3 Results

### 3.1 Patient characteristics

The cohort comprised 37 neurological WD patients (mean age 24.38 ± 6.40 years) and 37 healthy controls (25.08 ± 1.61 years), with no significant age difference between groups (*p* > 0.05). Table 1 summarizes all participants’ general and clinical features.

**Table 1:** General and clinical features of all participants.

Variables	WD patients (*n* = 37)	HCs (*n* = 37)
sex (Male/female)	20/17	22/15
age (years)	24.38 ± 6.40	25.08 ± 1.61
handedness	37 right-handed	37 right-handed
duration (years)	8.62 ± 5.95	–
UWDRS-N	10.19 ± 8.05	–
K-F ring (+/−)	30/7	–
Cp	<200 mg·L^−1^	–
U-Cu	>100 μg/24-h	–

WD, Wilson disease; HC, healthy controls; UWDRS-N, the Unified Wilson Disease Rating Scale for Neurology; K-F ring, Kayser-Fleischer corneal ring; Cp, serum ceruloplasmin; U-Cu, urinary copper.

### 3.2 GV differences between groups

**Figure 1:**
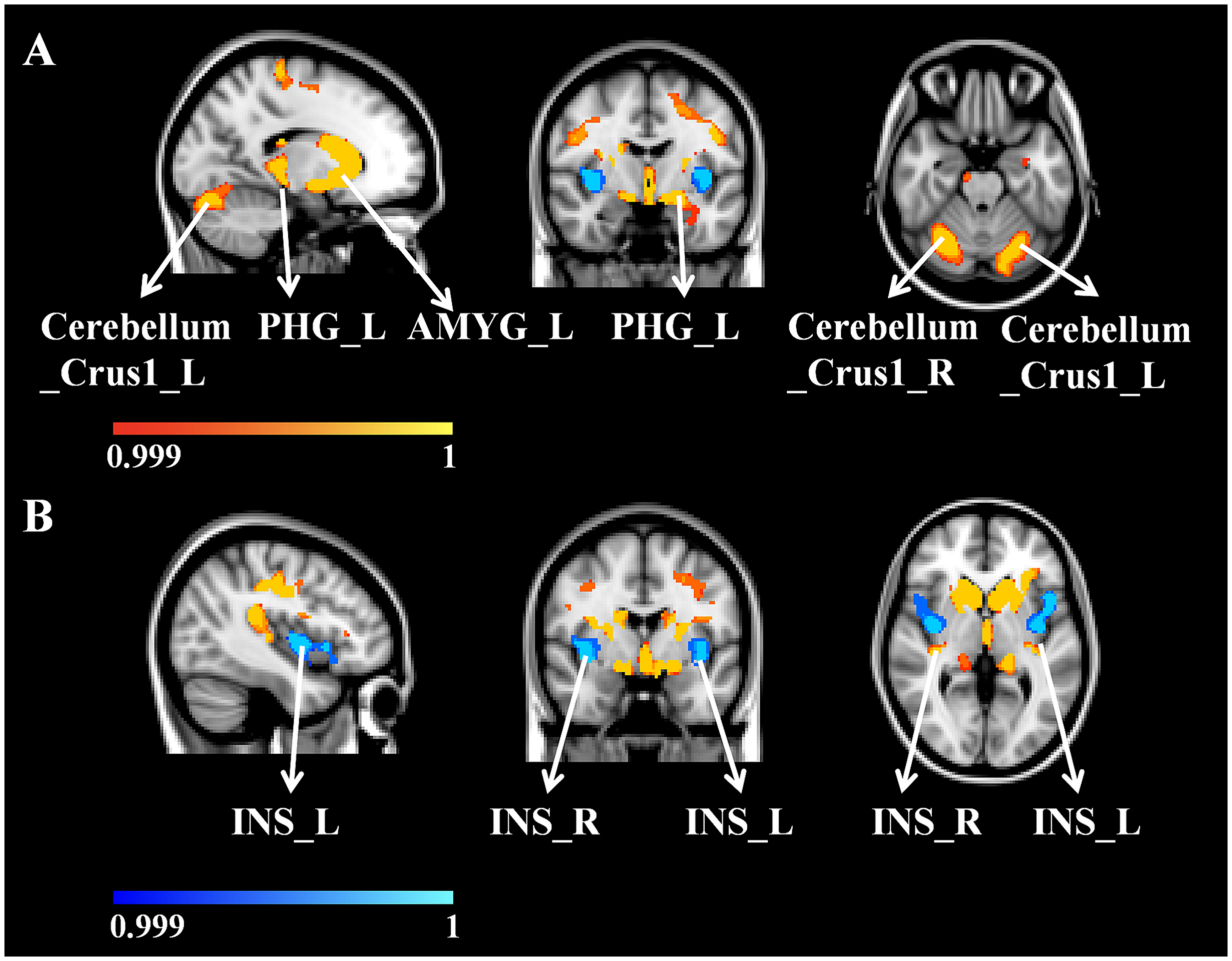
**(A)** WD patients GV decreased compared with HC. **(B)** WD patients’ GV increased compared with HC. WD, Wilson disease; HC, healthy controls; GV, gray matter volume; L, left; R, right; PHG, parahippocampal gyrus; AMYG, amygdala; INS, insular lobe.

Patients with WD exhibited decreased GV in the bilateral caudate nucleus, putamen, cerebellum (Crus 1), left amygdala, right posterior insular lobe, and right parahippocampal gyrus compared with healthy controls (Figure 1A; Table 2). However, compared to healthy controls, patients with WD exhibited an increased GV in the bilateral anterior insular lobes (Figure 1B; Table 2).

**Table 2:** Group GV differences between HCs and WD patients.

Regions	MNI coordinate			*p* value peak
	x	y	z	
HC > WD				
Left amygdala	-24	−5	−19	0.9998
Left cerebellum (Crus1)	−28	−66	−40	0.9998
Right cerebellum (Crus1)	30	−66	−40	0.9998
Right posterior insular lobe	34	−23	1	0.9998
Left caudate nucleus	−14	10	9	0.9998
Right caudate nucleus	18	12	7	0.9998
Left putamen	−20	14	9	0.9998
Right putamen	16	13	−9	0.9998
Right parahippocampal gyrus	18	−28	−11	0.9996
WD > HC				
Left anterior insular lobe	−36	−2	−10	0.9998
Right anterior insular lobe	40	−6	−7	0.9998

The coordinates align with the peak effect observed within the cluster. The FWE-corrected 1-P value demonstrates significant differences between the two groups. WD, Wilson disease; HC, healthy controls.

### 3.3 Correlations between GV and UWDRS-N

**Figure 2:**
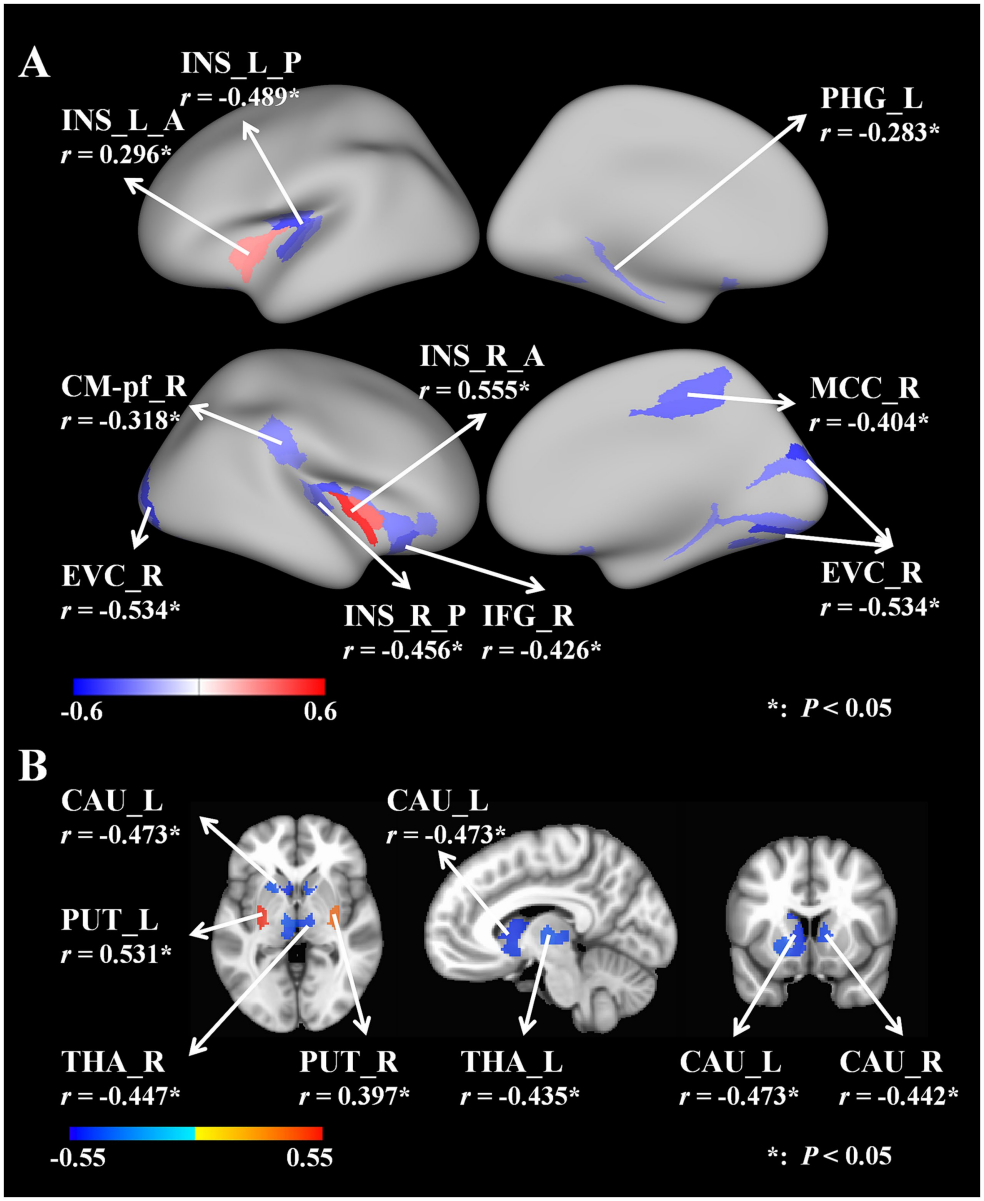
**(A)** Correlations between cortical areas’ GV and UWDRS-N. **(B)** Correlations between subcortical areas’ GV and UWDRS-N. UWDRS-N, the Unified Wilson Disease Rating Scale for Neurology; GV, gray matter volume; L, left; R, right; A, anterior; P, posterior; INS, insular lobe; PHG, parahippocampal gyrus; CM-Pf, parafascicular complex; EVC, early visual cortex; IFG, inferior frontal gyrus; MCC, mid-cingulate cortex; CAU, caudate nucleus; PUT, putamen; THA, thalamus.

In the cortical areas, we found that the GV from the bilateral anterior insular lobes (L, *r* = 0.296, *p* = 0.044; R, *r* = 0.555, *p* = 0.006) significantly correlated with the UWDRS-N. The GV from the posterior bilateral insular lobe (L, *r* = −0.489, *p* = 0.012; R, *r* = −0.456, *p* = 0.019); left parahippocampal gyrus (*r* = −0.283, *p* = 0.049); right parafascicular complex (*r* = −0.318, *p* = 0.044); right early visual cortex (*r* = −0.534, *p* = 0.006); right inferior frontal gyrus (*r* = −0.426, *p* = 0.024) and right mid-cingulate cortex (*r* = −0.404, *p* = 0.025) were also negatively correlated with the UWDRS-N (Figure 2A). Supplementary Table 1 provided a detailed account of the relevant data.

In the subcortical areas, we found that the GV from the bilateral putamen (L, *r* = 0.531, *p* = 0.034; R, *r* = 0.397, *p* = 0.034) was significantly positively correlated with the UWDRS-N. The GV from the bilateral caudate nucleus (L, *r* = −0.473, *p* = 0.041, R, *r* = −0.442, *p* = 0.041) and bilateral thalamus (L, *r* = −0.435, *p* = 0.041; R, *r* = −0.447, *p* = 0.041) were also found negatively correlate with the UWDRS-N. The brain images were generated using the Connectome Workbench and FreeSurfer version 6.0 (Figure 2B). Supplementary Table 2 provided a detailed account of the relevant data.

### 3.4 Correlations between GV and muscle tension value

**Figure 3:**
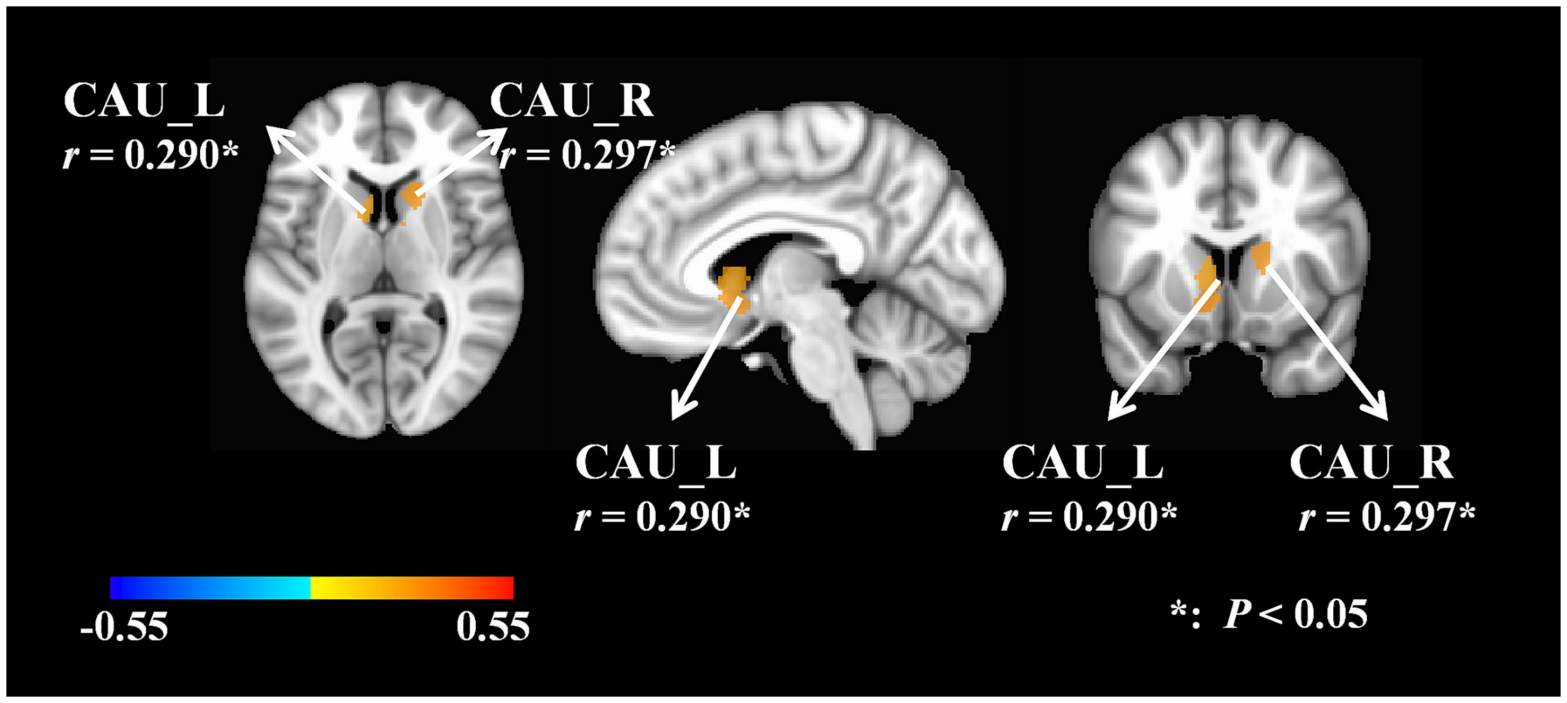
Correlations between GV and *F* value. F, frequency of muscle oscillation; L, left; R, right; CAU, caudate nucleus.

A positive correlation was observed between the GV of the bilateral caudate nucleus and the *F* value of the right gastrocnemius medial head (L, *r* = 0.290, *p* = 0.048; R, *r* = 0.297, *p* = 0.049). The brain images were generated with FreeSurfer version 6.0. The supplementary information provided a detailed account of the relevant data (Figure 3). Supplementary Table 3 provided a detailed account of the relevant data.

## 4 Discussion

In this study, we based on MRI data employed the VBM method to evaluate impaired GV in WD patients and correlate it with UWDRS-N and muscle tension levels.

The results show that WD patients with neurological symptoms have characteristic GV patterns compared to controls, which is analogous to reports from other studies (27). These areas are crucial for motor regulation and are primarily situated within the cortico-striatal-thalamo-cortical (CSTC) motor circuit, analogous to the findings of previous primary dystonia pathophysiology studies (28). It is postulated that the observed volume alterations are associated with neural compensatory mechanisms that emerge during the disease progression. The putamen, caudate nucleus, subthalamic nucleus, and cerebellum constitute the extrapyramidal system, in which the caudate nucleus plays a vital role in stabilizing random movements, maintaining muscle tension, and regulating limb posture (29). The atrophy of the cerebellum may be attributed to copper deposition within the nuclei, which exerts a toxic effect on cortical nerve cells (30). The bidirectional GV changes (atrophy/hypertrophy) observed in WD patients may reflect distinct temporal stages of neuropathology. Early copper deposition in subcortical regions could trigger GV increases, as seen in the anterior insula and putamen. This compensatory hypertrophy may arise from astrocyte proliferation or inflammatory responses to initial copper toxicity (31). Conversely, chronic copper accumulation induces neuronal loss and progressive atrophy in the thalamus and posterior insula, aligning with advanced disease stages (32). Such early compensation followed by late degeneration biphasic dynamics could explain regional variations in GV-symptom correlations. For example, preserved caudate volume which positively linked to muscle tension may represent early-stage inflammatory responses, while caudate atrophy which negatively associated with UWDRS-N reflects irreversible neuronal damage in later stages.

The positive correlation between caudate GV and right gastrocnemius muscle tension contrasting its negative association with UWDRS-N, suggests a dual role for caudate function (33). Preserved caudate volume may transiently elevate muscle tension via excessive glutamatergic drive to brainstem motor nuclei, a process potentially exacerbated by copper-mediated potentiation of glutamatergic signaling (34). While progressive caudate atrophy, however, reflects irreversible neuronal loss, decoupling structural integrity from symptom severity and driving composite UWDRS-N deterioration. The insular lobe is situated in a deep position within the lateral sulcus of each hemisphere, concealed beneath the frontal, parietal, and temporal lobes. Anatomically, the insular lobe is divided into the anterior and posterior sections by the central insular sulcus. Its atrophy may be associated with elevated blood ammonia and heavy metal deposition (35). The insular lobe is the convergence point for bodily sensation, autonomic control, and afferents from brain regions associated with emotional processing, such as the amygdala (36). The anterior dorsal insular consists of the dorsal portion of the anterior short insular gyrus and the middle short insular gyrus, which are involved in processing cognitive tasks. The posterior portion of the insular consists of the long and short insular gyrus, which are involved in processing sensorimotor tasks (37). Early studies of the macaque insula structural connections showed that direct cortical stimulation of the insular region resulted in a range of involuntary movements, including those of the face, body, and tail. This stimulation led to notable respiration, heart rate, blood pressure, and salivation changes. The results indicate the existence of direct structural connections between the insula, the motor cortex, and the autonomic nervous system (38). A magnetic resonance diffusion-weighted imaging study conducted in human subjects identified anterior–posterior differences in structural connectivity comparable to those observed in the macaque insular region (39). This evidence indicates that structures such as the striatum, cerebellum, and insula have significant functions in regulating neural processes.

The correlation between UWDRS-N and the brain regions exhibiting variability was analyzed which revealed that elevated GV of the bilateral anterior insular lobes in the cortical areas showed a significant positive correlation with UWDRS-N. Additionally, GV in the bilateral posterior insular lobes, left parahippocampal gyrus, right parafascicular complex, right early visual cortex, right inferior frontal gyrus, and right middle cingulate cortex showed a negative correlation with UWDRS-N. These findings show that increased GV in the bilateral anterior insula lobes is associated with more severe neurological symptoms. In contrast, decreased GV in the posterior insula lobes, part of the temporal lobe, optic cortex, frontal lobe, and cingulate cortex is associated with less severe neurological symptoms. A significant positive correlation was found between GV from the bilateral putamen and UWDRS-N in the subcortical areas. In contrast, the bilateral caudate nucleus and thalamus showed a significant negative correlation. This indicates that increased bilateral putamen GV is associated with more severe neurological symptoms. In contrast, decreased bilateral caudate nucleus and thalamus GV are associated with less severe neurological symptoms.

Dystonia is one of the most common movement disorders in WD, along with tremor, Parkinsonism, and ataxia. Moreover, dystonia is widely recognized as the most challenging movement disorder to manage in WD patients (21). Patients may exhibit prodromal symptoms in the initial disease stages, such as mild ataxia and involuntary finger movements. As the disease progresses, patients may develop more severe movement disorders, including torsion spasm, tremor, and chorea. These symptoms can significantly impact daily life and the ability to work (40). Significant positive correlation was observed between GV in the bilateral caudate nucleus and the right gastrocnemius medial head muscle tension in patients with WD. In general, WD dystonia is more commonly associated with the putamen (41), particularly in paediatrics (42). However, this was not observed in our study, potentially due to the inclusion of a predominantly adult patient cohort.

It was therefore speculated that patients with WD who present with neurological symptoms, particularly dystonia, may be associated with CSTC circuit damage. Future research and treatment may seek to further enhance the damaged CSTC circuit of WD through transcranial magnetic stimulation (TMS) and other therapeutic methods to improve neurological symptoms. This provides a novel approach to addressing the neurological symptoms of WD.

Despite stringent methodological controls, this study has limitations. First, our final inclusion of 37 patients, although considerable for a rare disease cohort, may reduce statistical power to detect weaker correlations between neuroanatomical changes and clinical severity, particularly given the heterogeneous expression of dystonia in WD. Second, the observational cohort design focused on natural disease progression without evaluating therapeutic interventions. Future longitudinal studies could assess the effects of pharmacological treatments or other modalities on neuroanatomical trajectories in WD. Additionally, multi-center collaborations are warranted to validate these findings in larger, independent cohorts.

## 5 Conclusion

In this research, we found that patients with WD exhibiting neurological symptoms demonstrated distinct GV alterations within the CSTC circuit, including the anterior insula, caudate nucleus, and thalamus. These GV changes correlated with clinical neurological symptoms including dystonia. Finally, structural abnormalities in the CSTC circuit, particularly in the motor regulatory regions, may underlie the pathophysiology of the neurological symptoms of WD including dystonia.

## Data Availability

The original contributions presented in the study are included in the article/Supplementary material, further inquiries can be directed to the corresponding authors.
